# Supplementary material for: Telomere-to-telomere Phragmites australis reference genome assembly with a B chromosome provides insights into its evolution and polysaccharide biosynthesis
Source: Commun Biol. 2025 Jan 17;8:73. doi: 10.1038/s42003-025-07532-y (PMC11742667; doi:10.1038/s42003-025-07532-y)
Supplement: Supplementary file 1 — Supplementary information [file 42003_2025_7532_MOESM1_ESM.pdf]

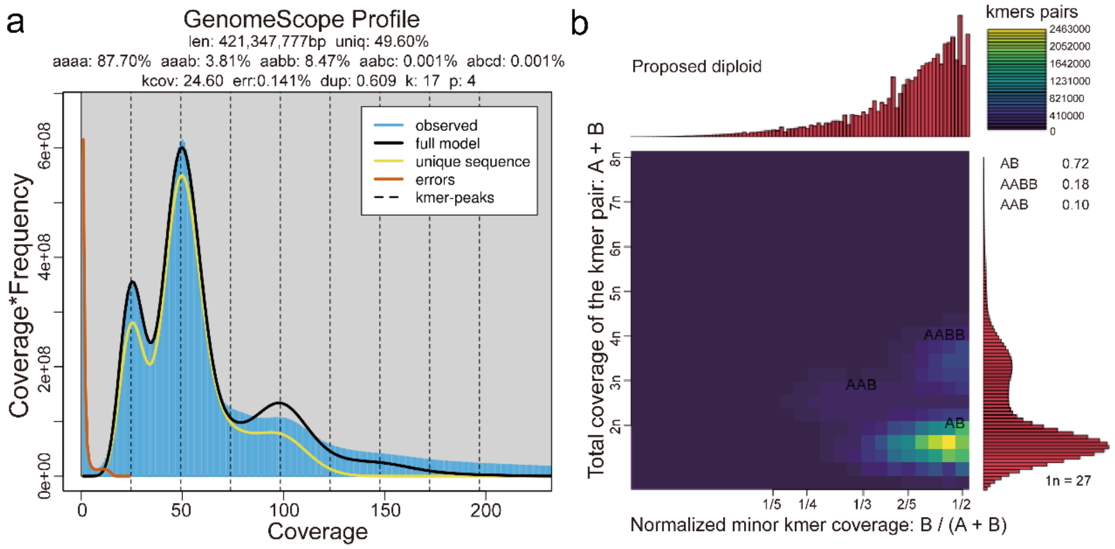

Supplementary Figure 1. Survey analysis of the *P. australis* genome. a The 17-mer depth and number frequency distribution. Analyzed and visualized using GenomeScope 2.0. **b** Smudgeplot of the *P. australis* genome with relative coverage (CovB / (CovA + CovB)) in the horizontal, total coverage (CovA + CovB) in the vertical, and colors indicating the frequency of k-mer pairs. Each possible haplotype structure is presented as a "Smudge" on the graph, and the heat of the "Smudge" indicates the frequency of the haplotype structure in the genome.

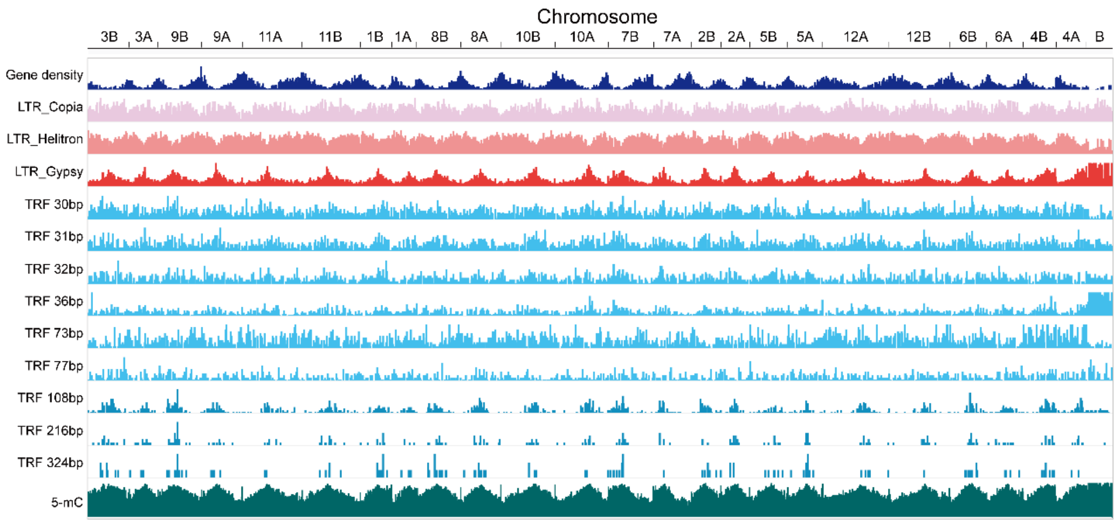

Supplementary Figure 2. The distribution of gene density, different types of TE and TRF, and 5-methylcytosine in the chromosomes. The distribution of these data was visualized using IGV.

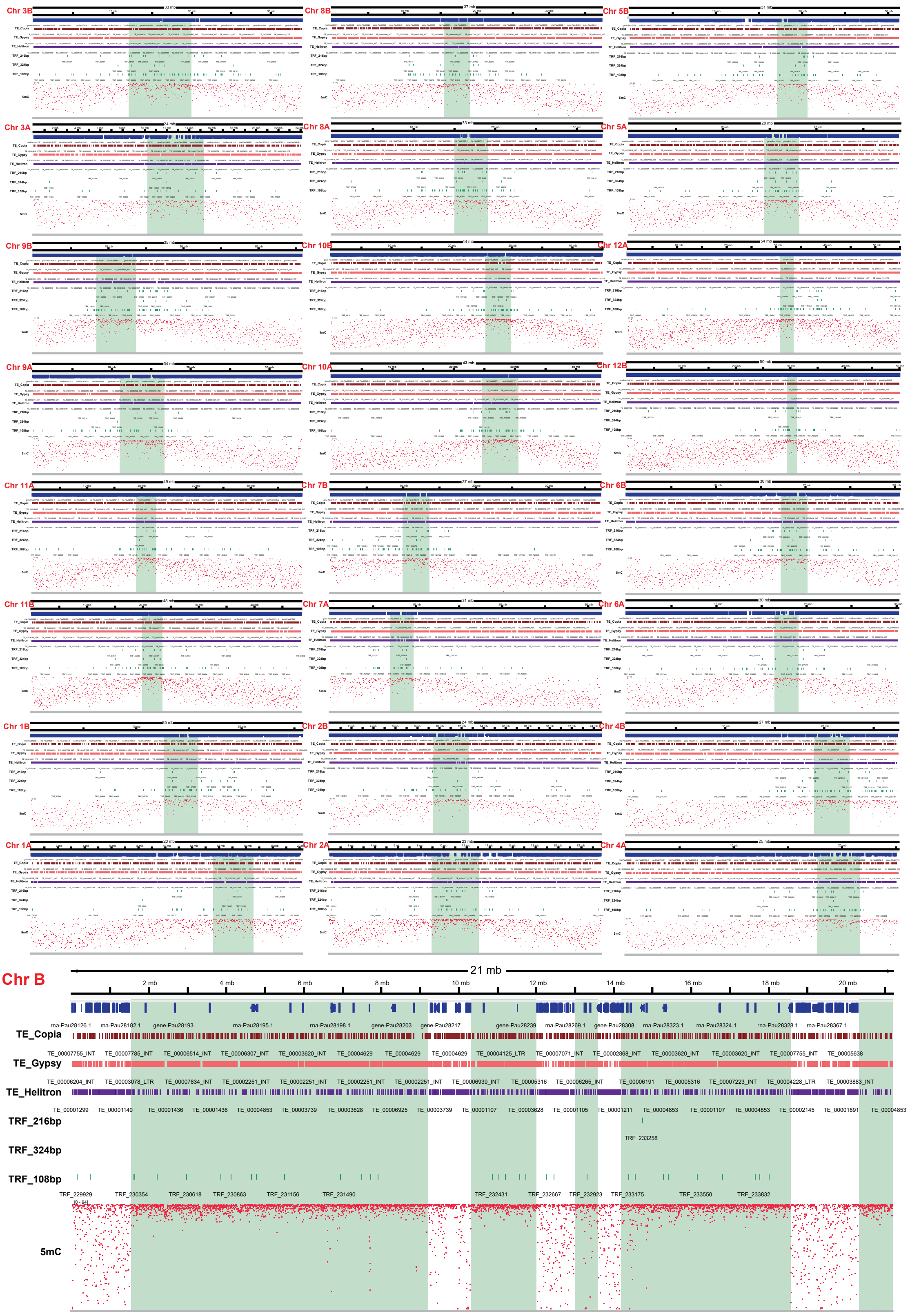

Supplementary Figure 3. 5-Methylcytosine distribution density distribution identifies candidate locations for *P. australis* centromere. The green background indicates candidate centromere locations.

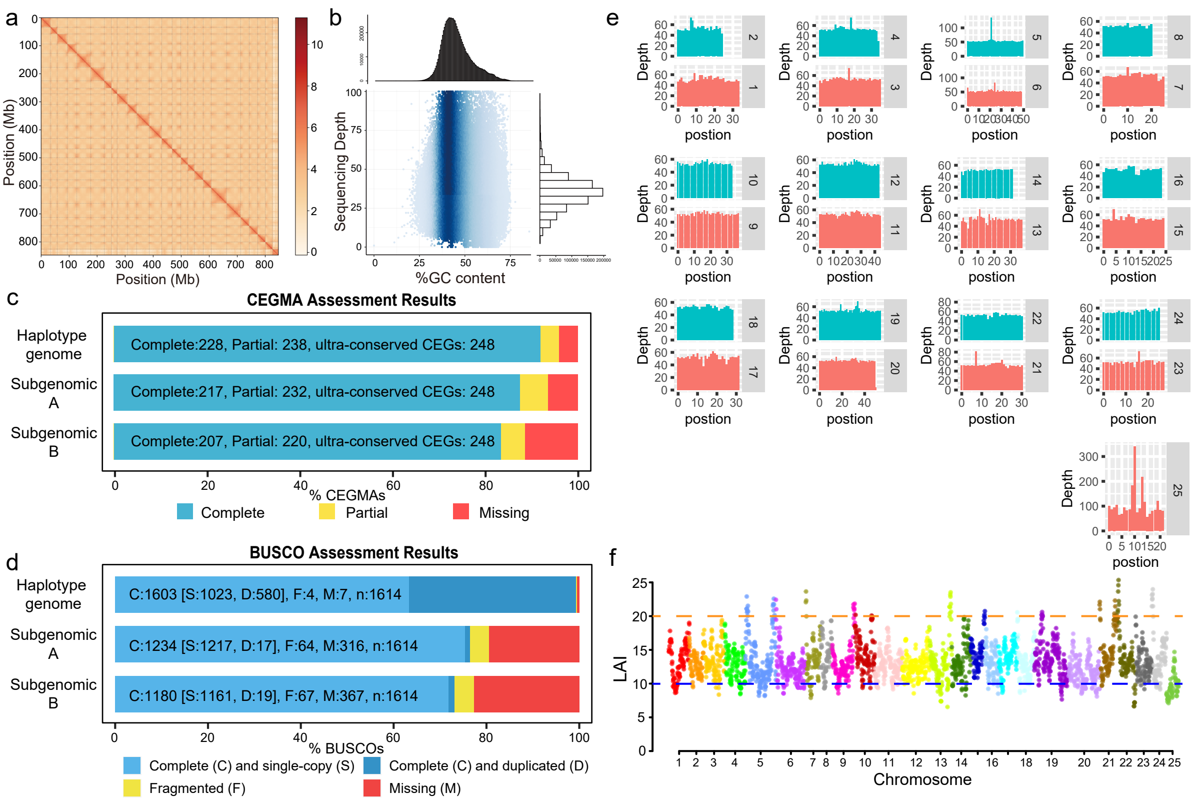

Supplementary Figure 4. Genome assembly quality assessment. a Genome-wide Hi-C interactions heatmap of the *P. australis* genome. Colors from light to dark in the plot indicate increasing strength of interactions, with darker colors showing stronger interactions. The 25 squares on the diagonal of the plot are the 25 chromosomes of *P. australis*, and there is no obvious clustering error (3C). **b** Density plot of GC content distribution and sequencing depth distribution in the genome of *P. australis*, with darker dots representing a higher number of dots here. **c** CEGMA assessment of gene region integrity in the genome. **d** BUSCO assessment. The BUSCO single-copy homozygous gene set is embryophyta_odb10. **e** Assessment of assembly integrity and uniformity of sequencing coverage by mapping second-generation data into the assembled genome, with the vertical coordinate indicating second-generation data coverage.**f** Genetic integrity on the 25 chromosomes assembled was assessed by LAI, with the horizontal coordinate indicating the chromosome number of the assembly and the vertical coordinate indicating the LAI of a particular chromosome.

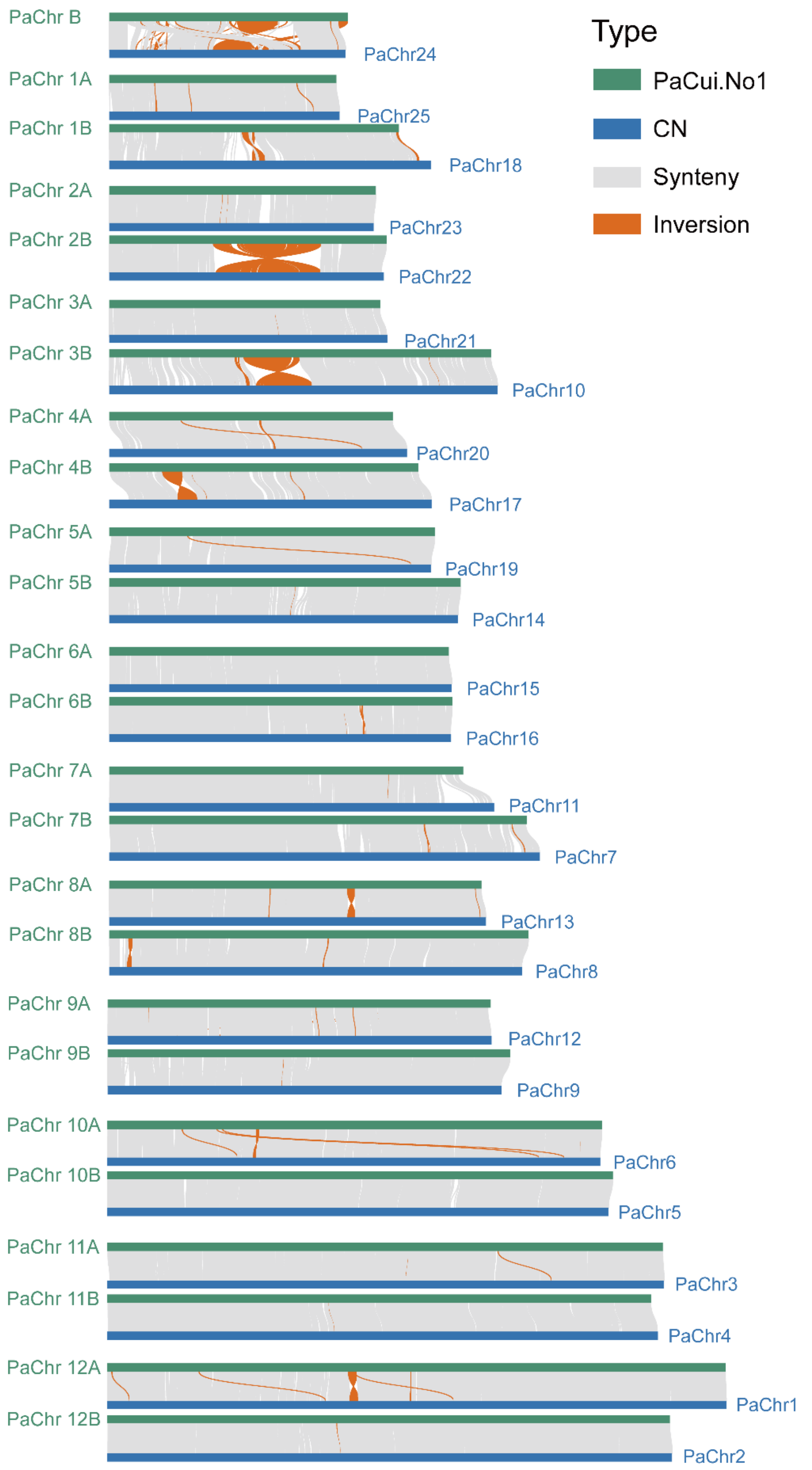

Supplementary Figure 5. The visualisation of the synteny and structural variations between the *P. australis* genomes PaCui.No1 (green) and CN (blue). and CN (blue).

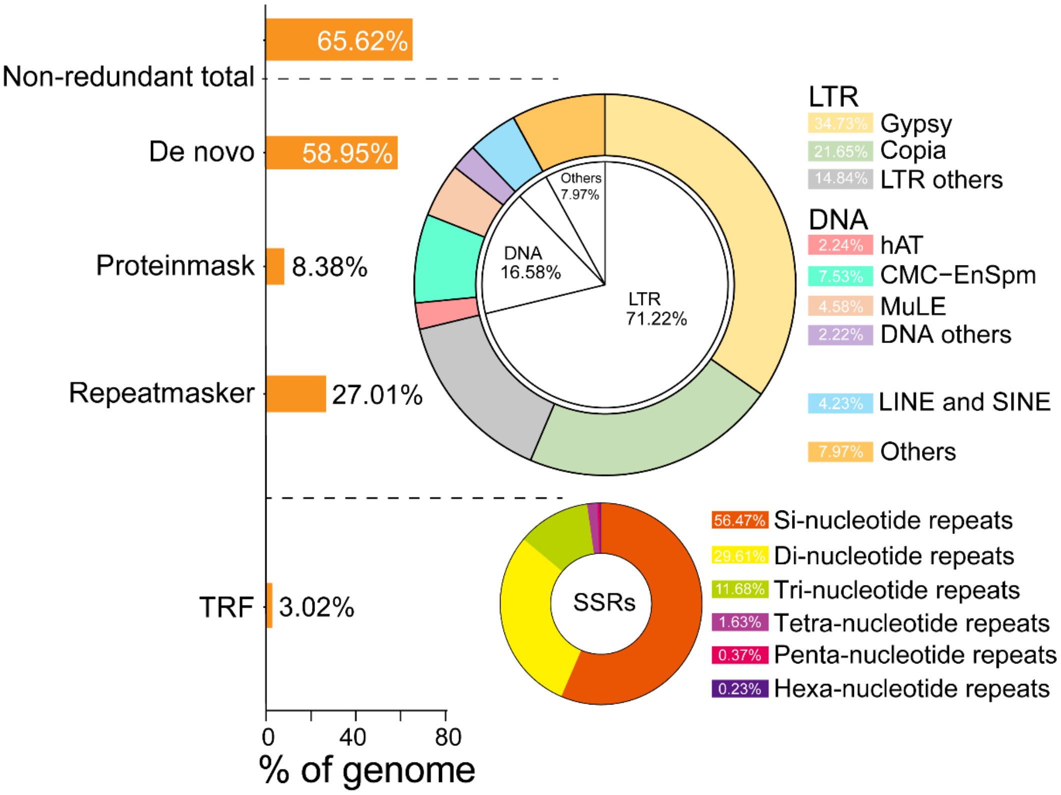

Supplementary Figure 6. Repetitive sequence annotation in the *P. australis* genome. The bar graph on the left indicates the results of repeated sequence prediction in the *P. australis* genome using different strategies.

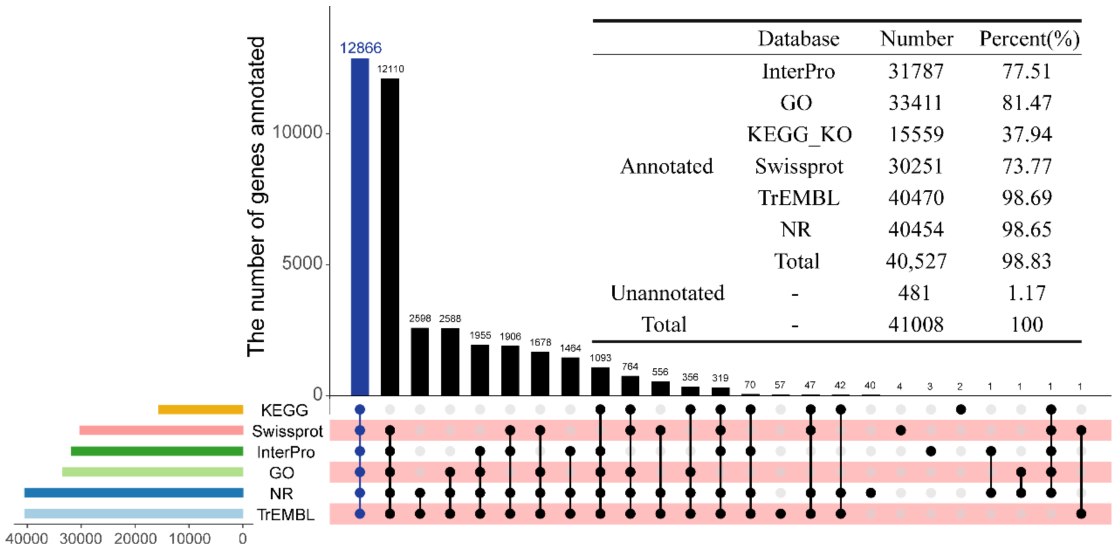

	Database	Number	Percent(%)
	InterPro	31787	77.51
	GO	33411	81.47
	KEGG_KO	15559	37.94
Annotated	Swissprot	30251	73.77
	TrEMBL	40470	98.69
	NR	40454	98.65
	Total	40,527	98.83
Unannotated	-	481	1.17
Total	-	41008	100

Supplementary Figure 7. Upset plots of the functional annotation of the *P. australis* genome. The blue bars in the figure indicate the number of genes for which the best matches exist in six databases simultaneously. Visualization was performed using the UpSetR (https://github.com/hms-dbmi/UpSetR) packages in R.

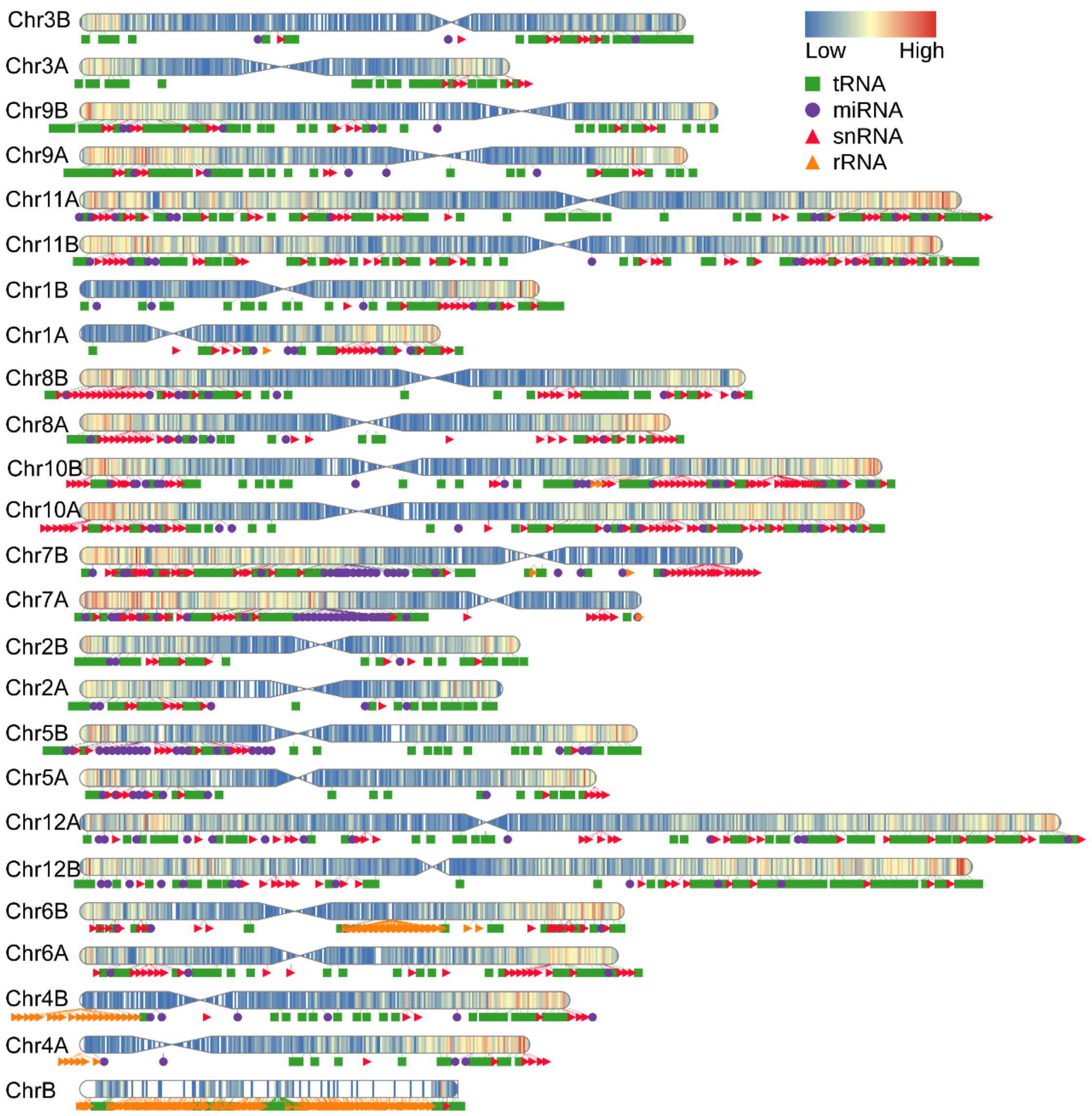

Supplementary Figure 8. Distribution of non-coding RNAs on chromosomes in the *P. australis* genome.

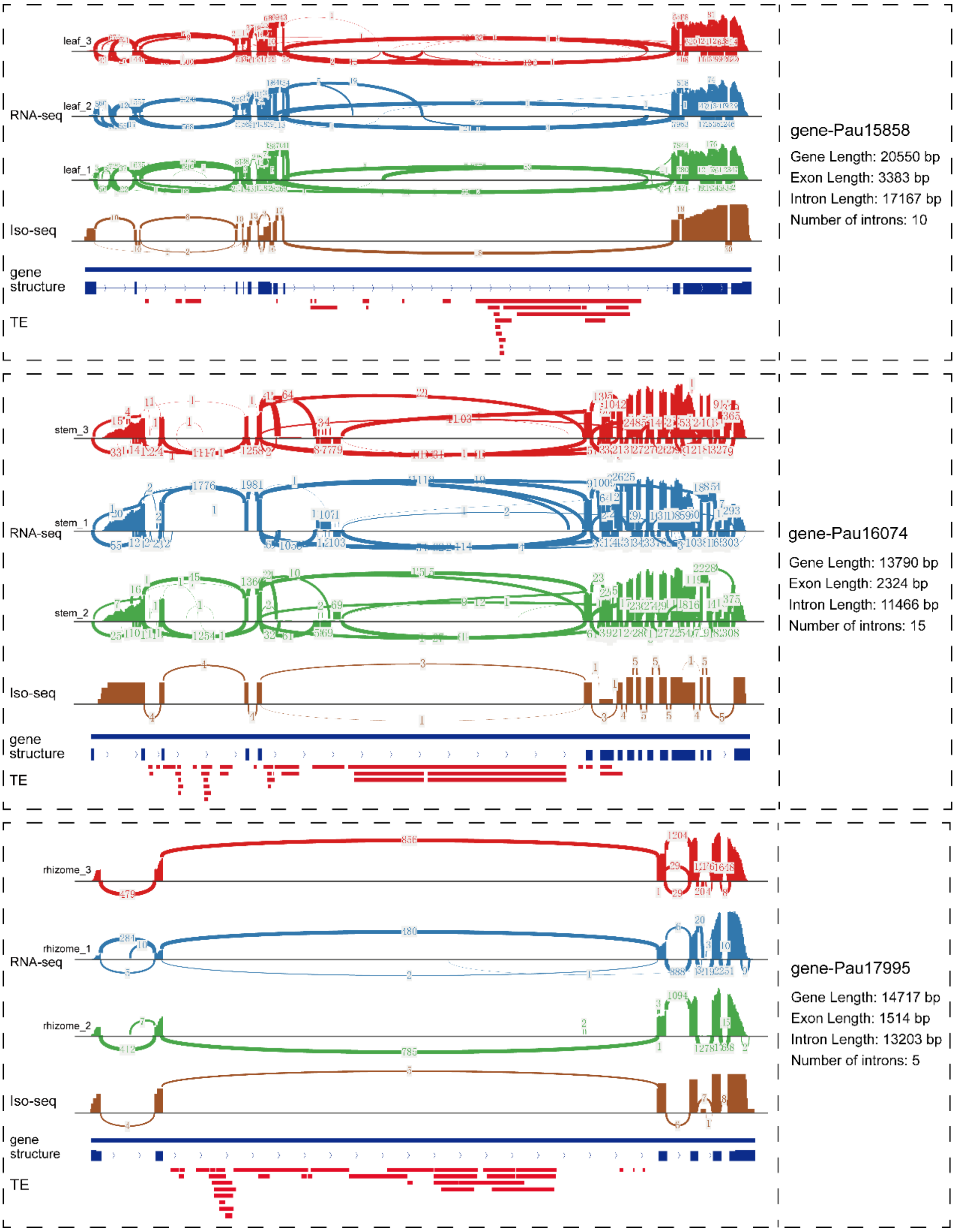

Supplementary Figure 9. Sashimi plot of reed tissue-specific expressed genes (with extra-long introns). RNA-Seq, Iso-Seq, gene structure, and TE distribution in genes are shown from top to bottom. The *P. australis* density of the sequencing data is shown as a histogram.

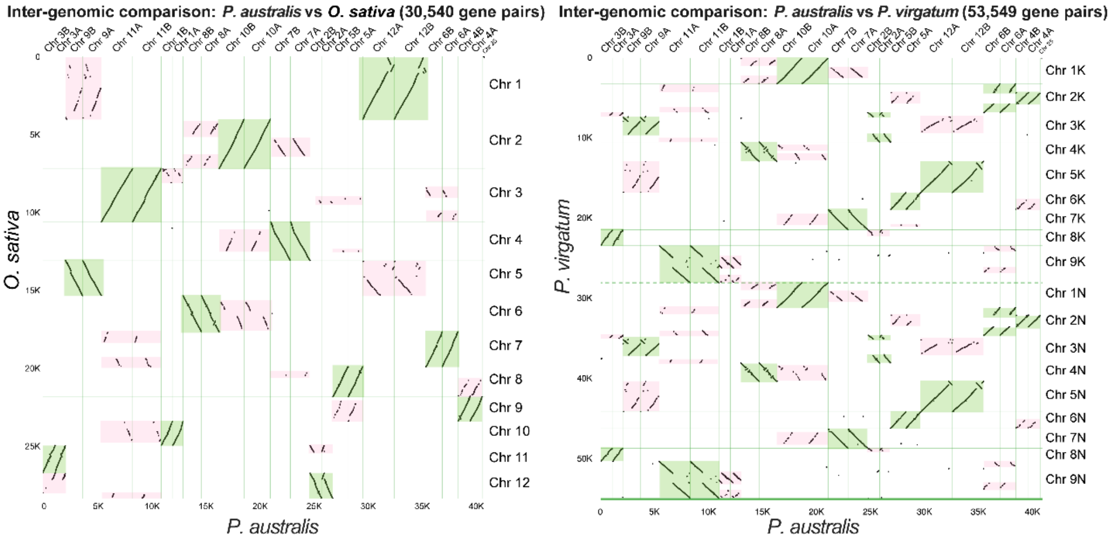

Supplementary Figure 10. Chromosomal synteny of *P. australis* with *Oryza sativa* and *Panicum virgatum*, respectively. All syntenic genes with C-score ≥ 0.7 are shown using the jcvi ortholog algorithm.

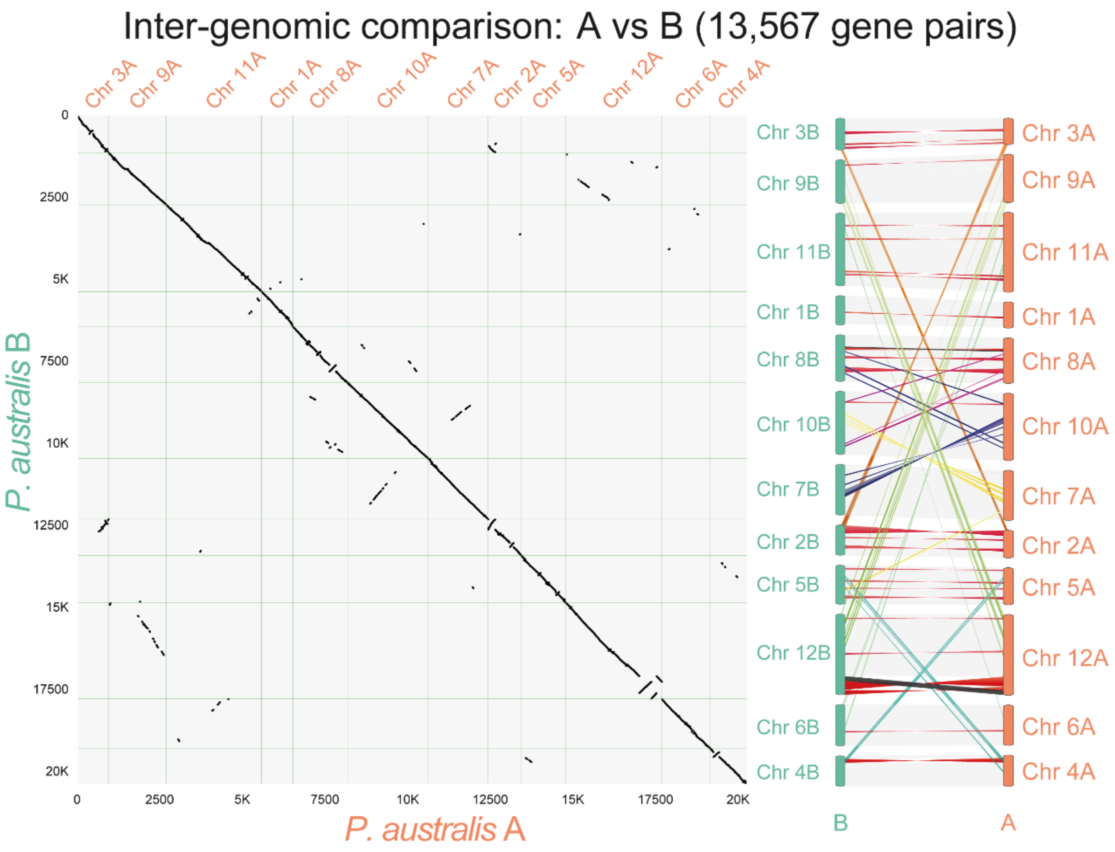

Supplementary Figure 11. Dot plot of colinearity analysis of the two subgenomes of *P. australis*. The right side indicates syntenic blocks between subgenomes A and B, and red crosshairs indicate chromosome inversions.

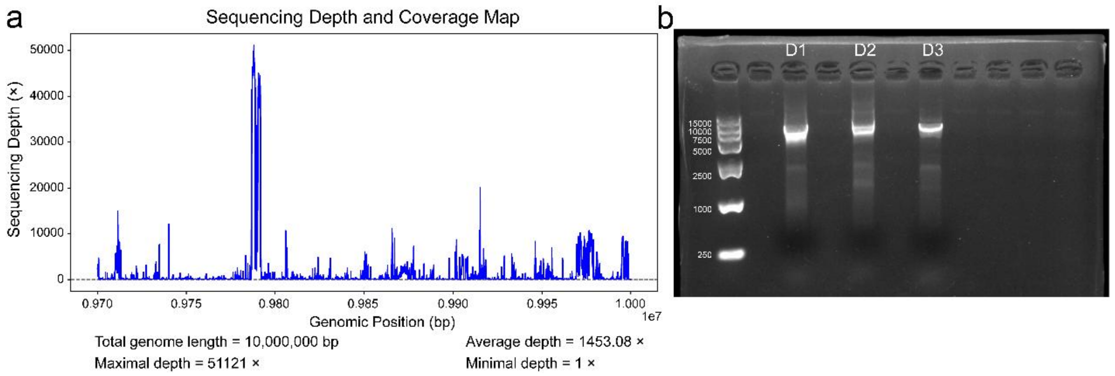

Supplementary Figure 12. Validation of a high-density region of chromosome B tRNA. a Sequencing depth and coverage within this region was measured by short-reads genomic sequencing data. **b** PCR validation of the continuity of tRNA high-density region assembly. We designed three overlapping primers within this tRNA high-density region and verified the assembly continuity of this region by PCR experiments. D1 - D3 primers are shown in Supplementary Table 7.

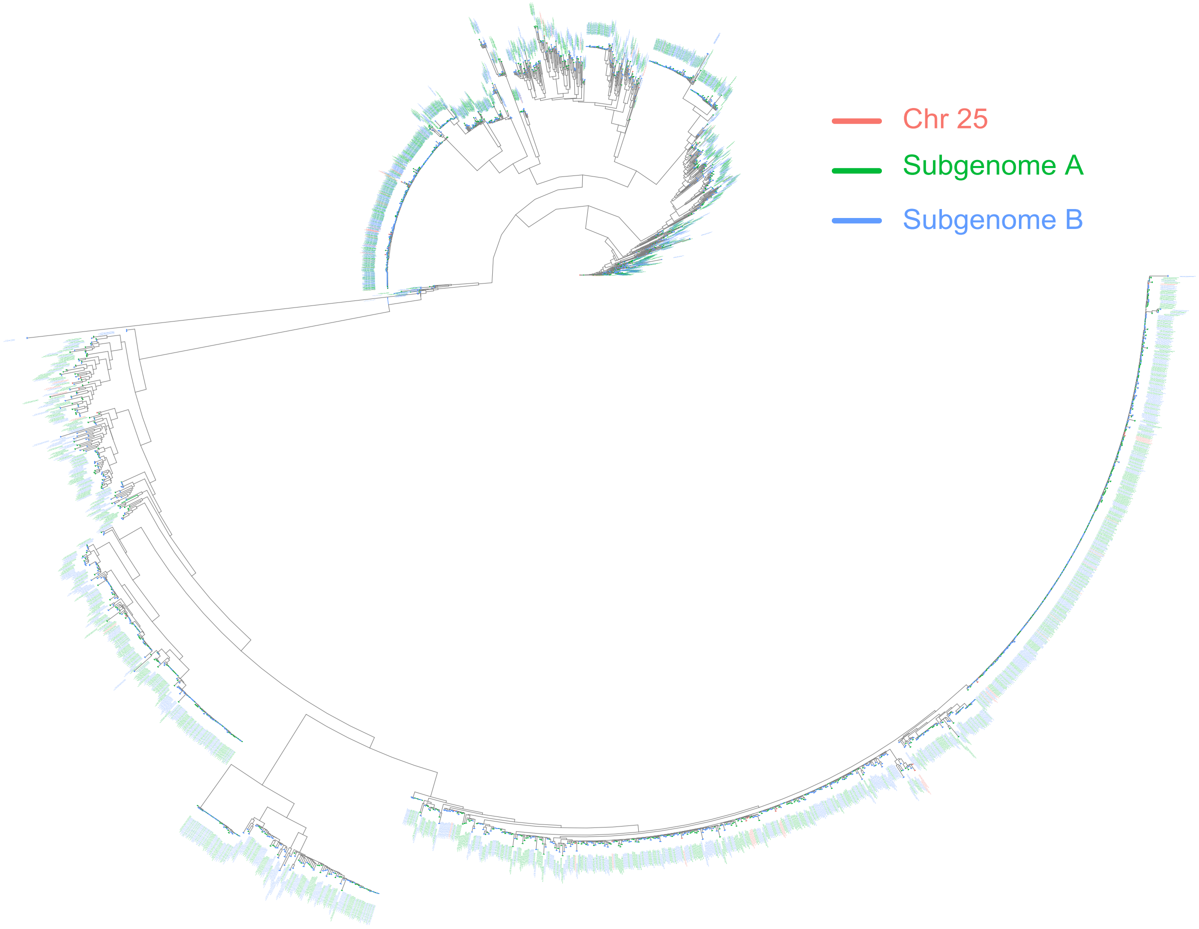

Supplementary Figure 13. Evolutionary analysis of the complete LTR-RT sequences of Chr25 and two sets of subgenomes. The phylogenetic tree was constructed using the maximum likelihood (ML) method with iqtree (v 2.1.4) software. The model used was JTT+F+R9 with 1000 bootstrap replicates.The LTR-RT sequences are distinguished by different colours in the phylogenetic tree; further details can be found in the treefile (https://doi.org/10.6084/m9.figshare.27016279).

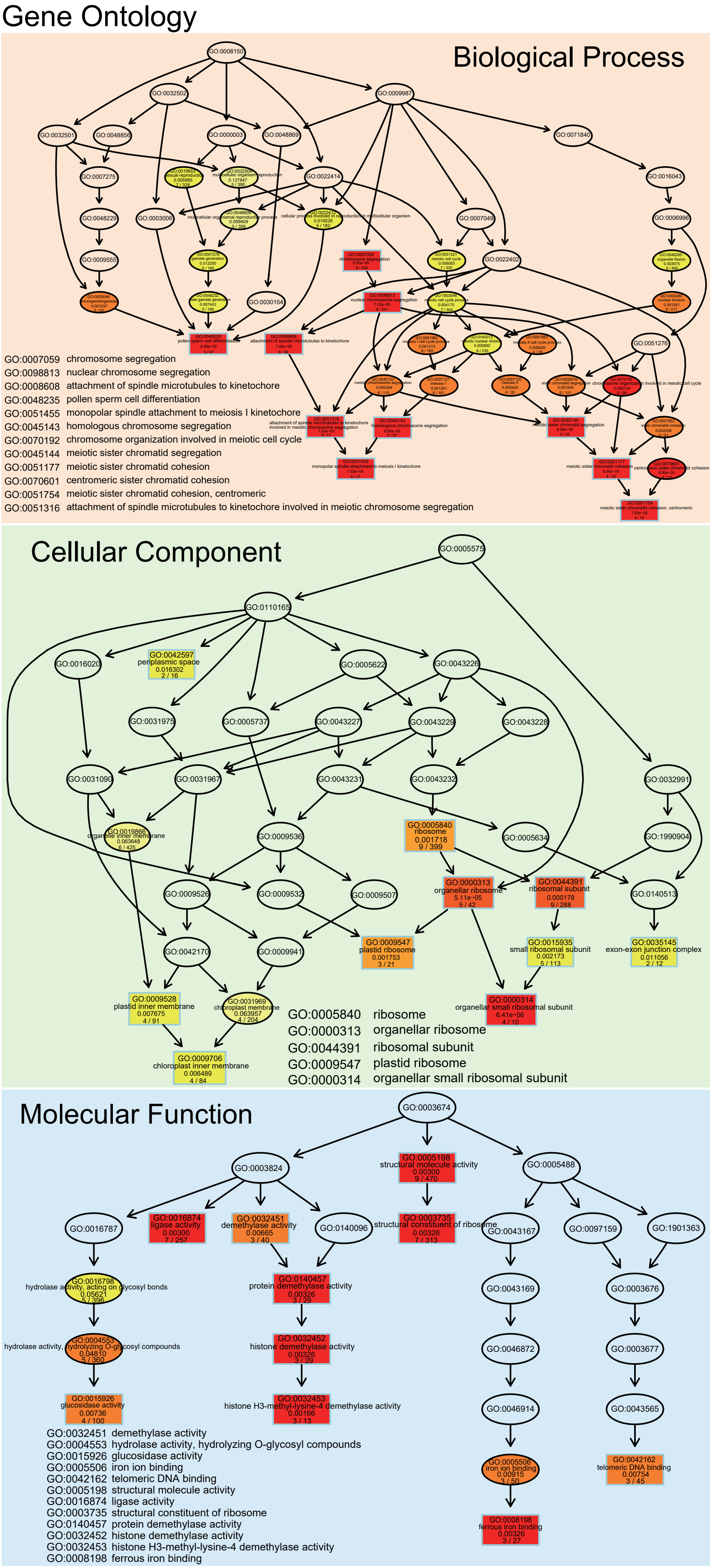

Supplementary Figure 14. Directed acyclic map of GO enrichment of genes in the *P. australis* B chromosome. The more specific the molecular function, the lower the hierarchy. The ten most significant nodes are indicated by the boxes in the Supplementary Figure. The enrichment significance of each node is differentiated by color, from highest to lowest: red > orange > yellow.

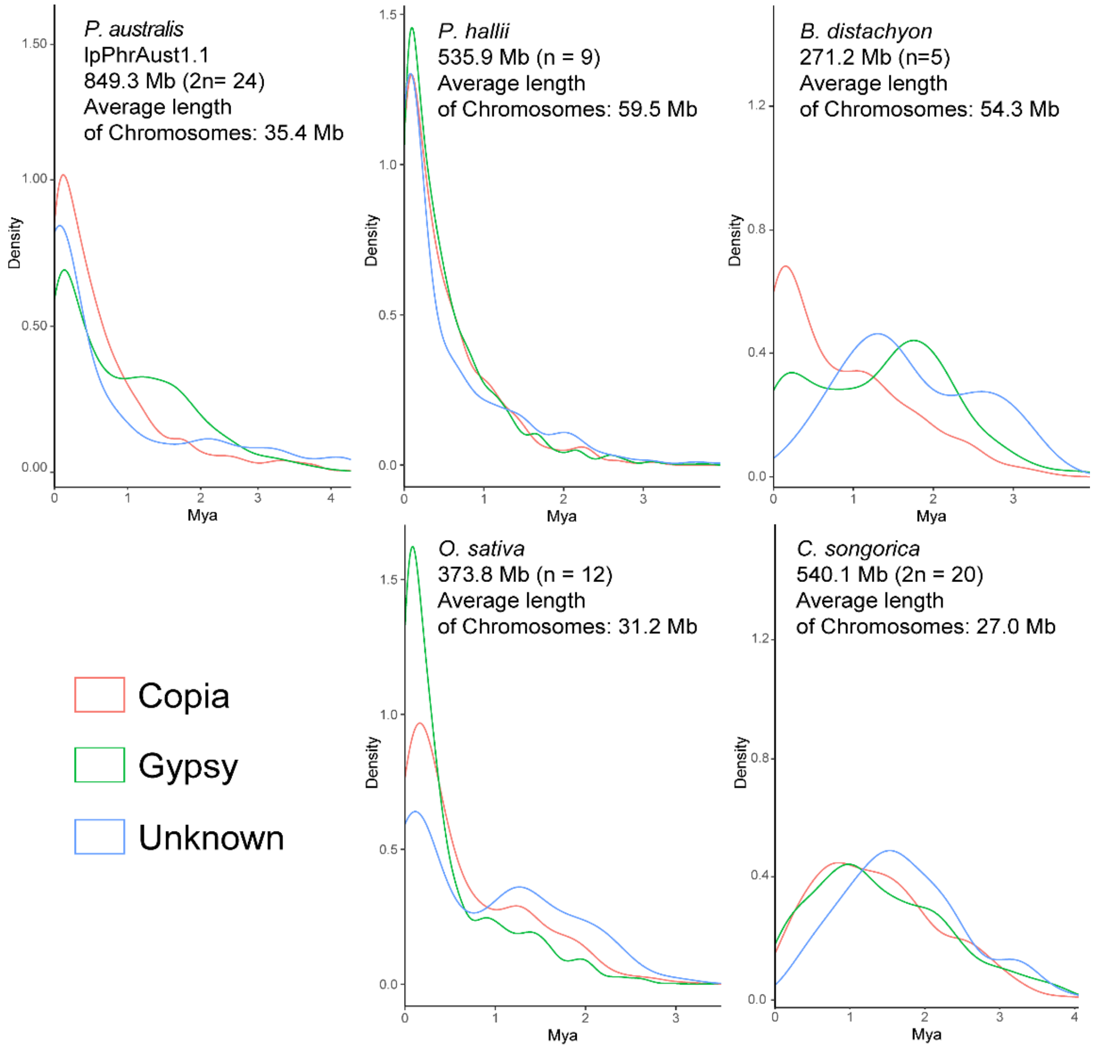

Supplementary Figure 15. Distribution of insertion densities of different types of LTR-RTs in five Graminae plants. Including the *P. australis* (IpPhrAust1.1), *Panicum hallii, Brachypodium distachyon, Oryza sativa,* and *Cleistogenes songorica.*

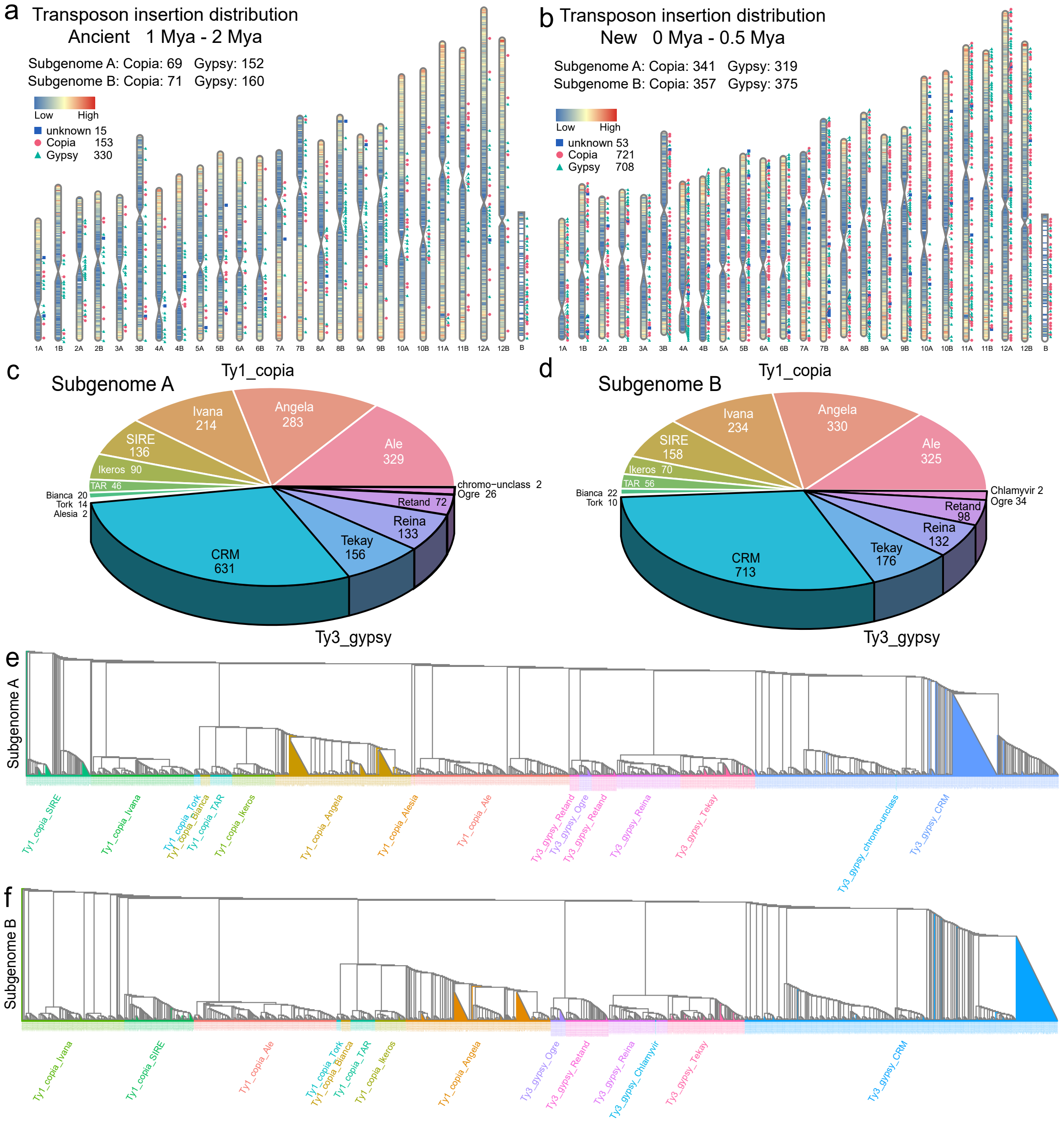

Supplementary Figure 16. Analysis of transposon insertion events in the *P. australis* genome. a-b Distribution of ancient (a) and new (b) inserted LTR-RTs on chromosomes. **c-d** Copia and Gypsy subfamily type counts in *P. australis* subgenomes A and B. **e-f** Evolutionary analysis of Copia and Gypsy sequences in *P. australis* subgenomes A and B. Evolutionary tree construction based on complete reverse transcriptase domain sequences in LTR-RTs sequences and visualisation with ggtree (R package). The LTR-RT sequences are distinguished by different colours in the phylogenetic tree; further details can be found in the treefile (https://doi.org/10.6084/m9.figshare.27016279).

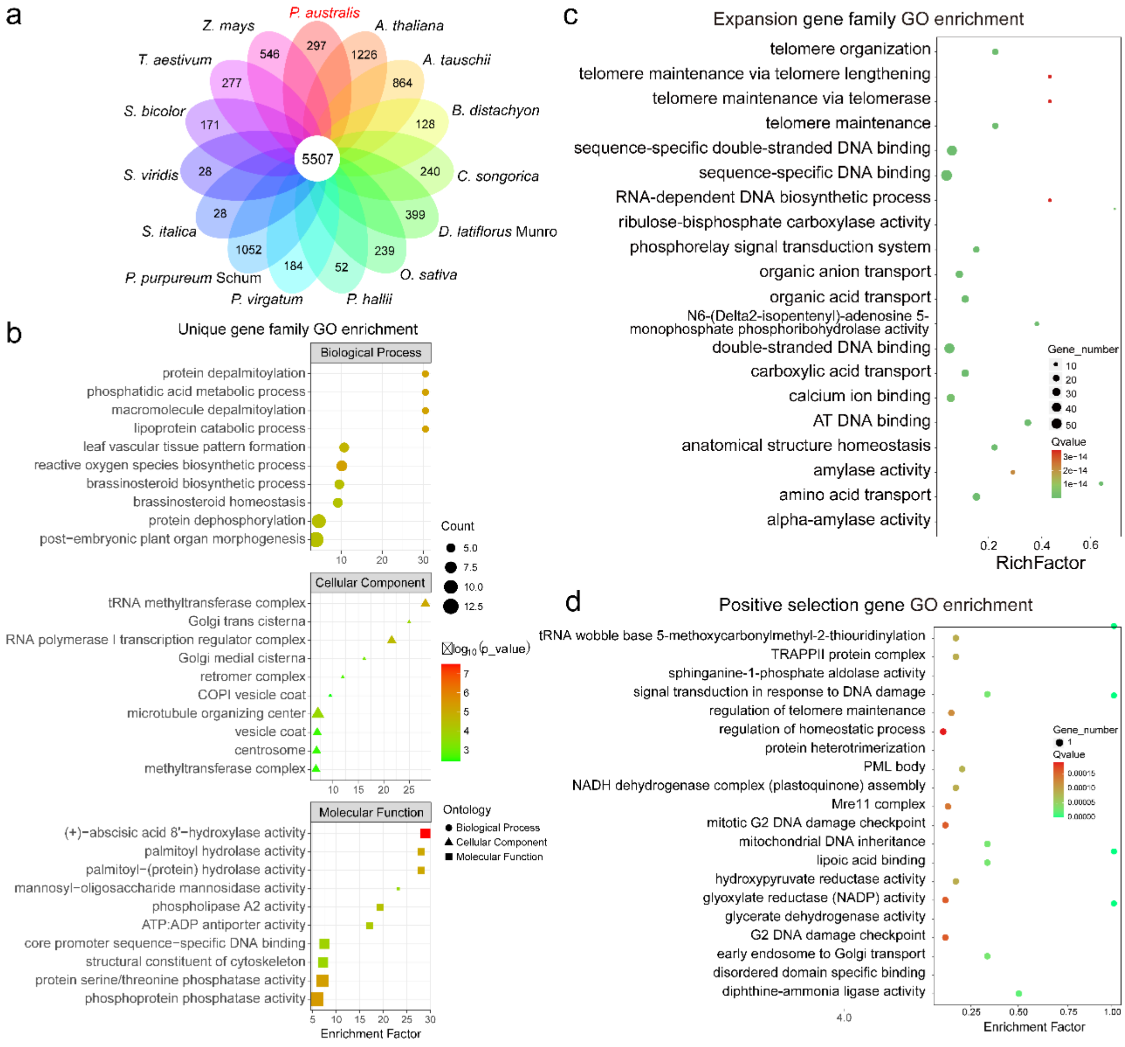

Supplementary Figure 17. *P. australis* gene family enrichment analysis. a Venn diagram of 15 species sharing or unique gene family clustering. The numbers in the diagram indicate the number of species gene families. **b** GO enrichment analysis of individual gene families in *P. australis*. **c** GO enrichment analysis of gene family expansions occurring in *P. australis*. **d** GO enrichment of the positive selection genes in *P. australis*.

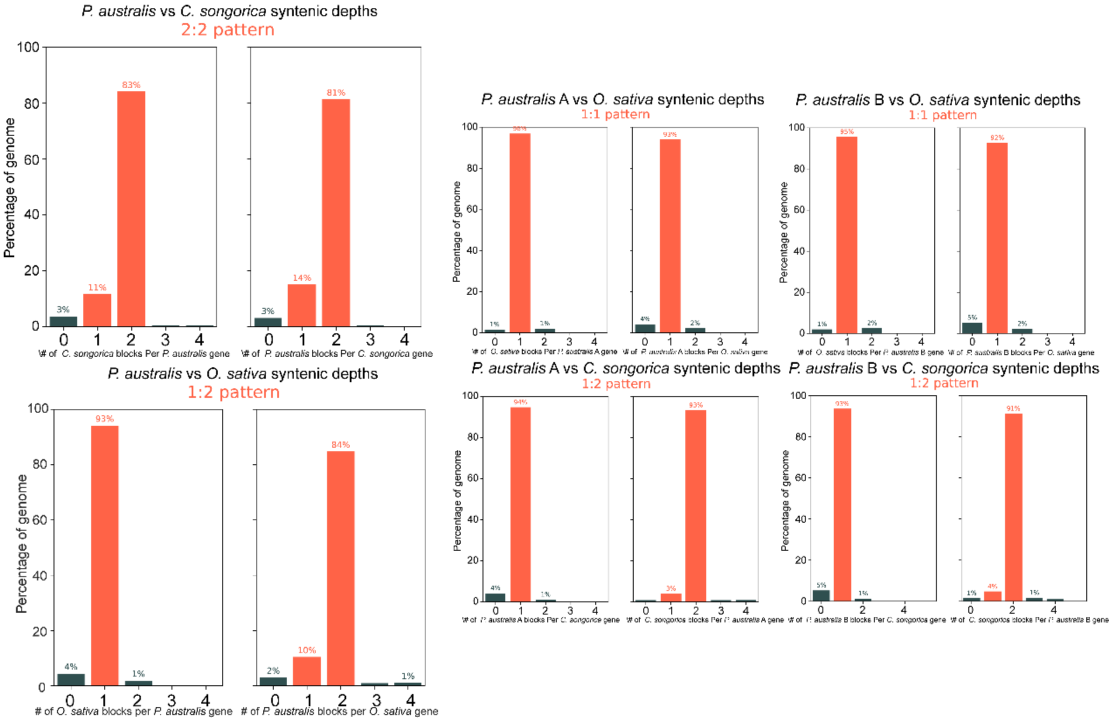

Supplementary Figure 18. The syntenic depth of *P. australis* genomes/subgenomes with *Cleistogenes songorica* and *O. sativa*, respectively.

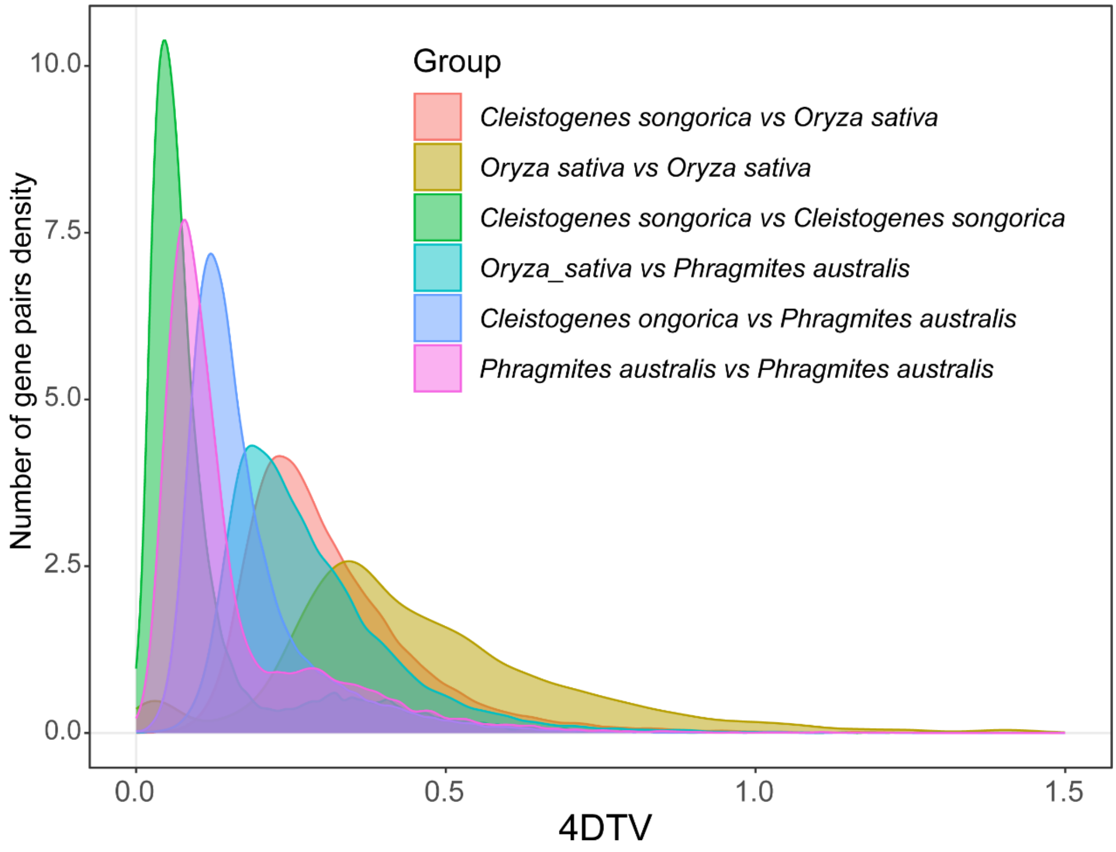

Supplementary Figure 19. Distribution of Four-fold synonymous third-codon transversion rate (4Dtv) distances for gene pairs in the syntenic blocks of *P. australis*, *O. sativa*, and *C. songorica* genomes.

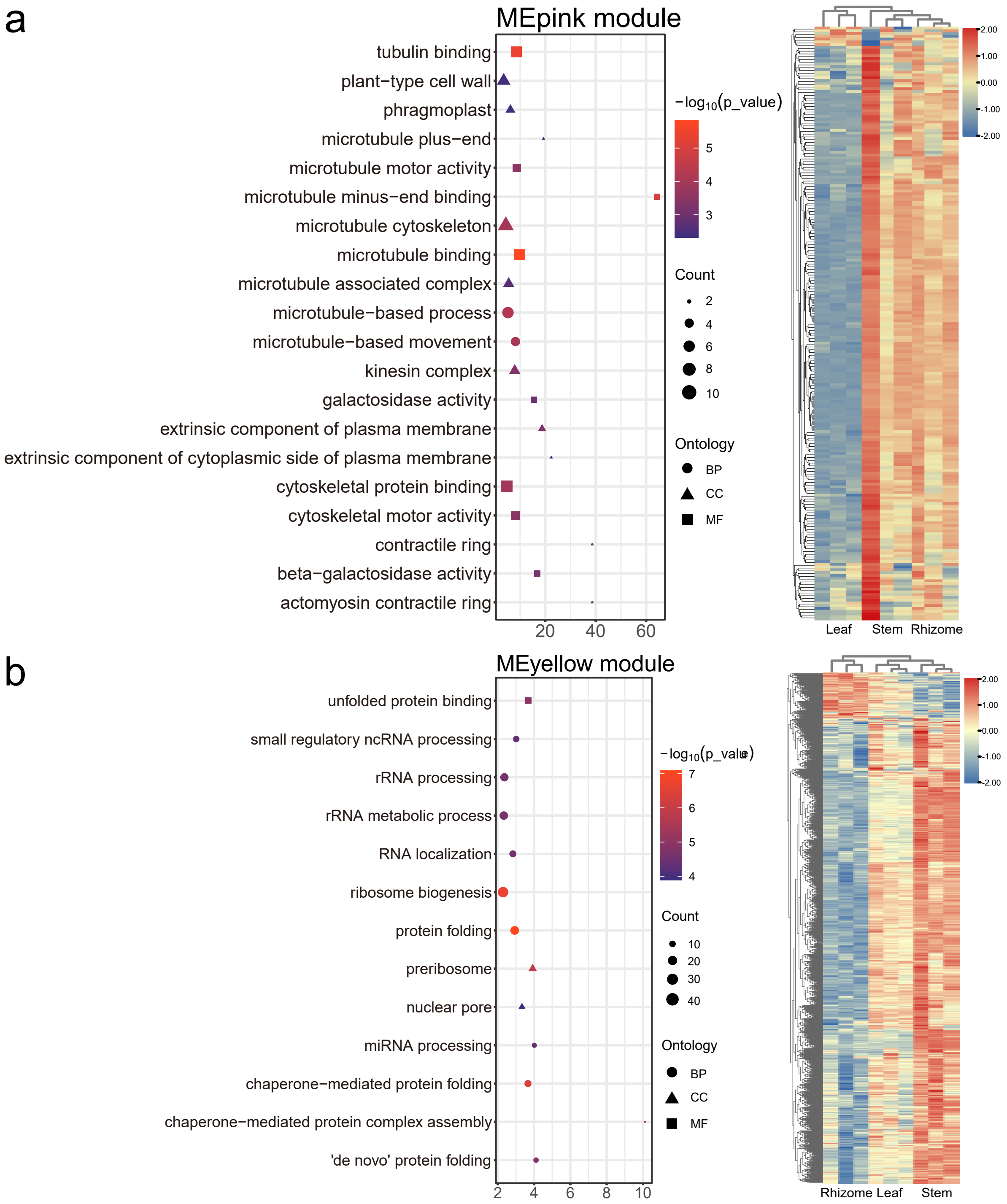

Supplementary Figure 20. GO enrichment results of genes in the MEpink (a) and MEyellow (b) modules and changes in module eigengene expression (FPKM) after row-scaling treatment.

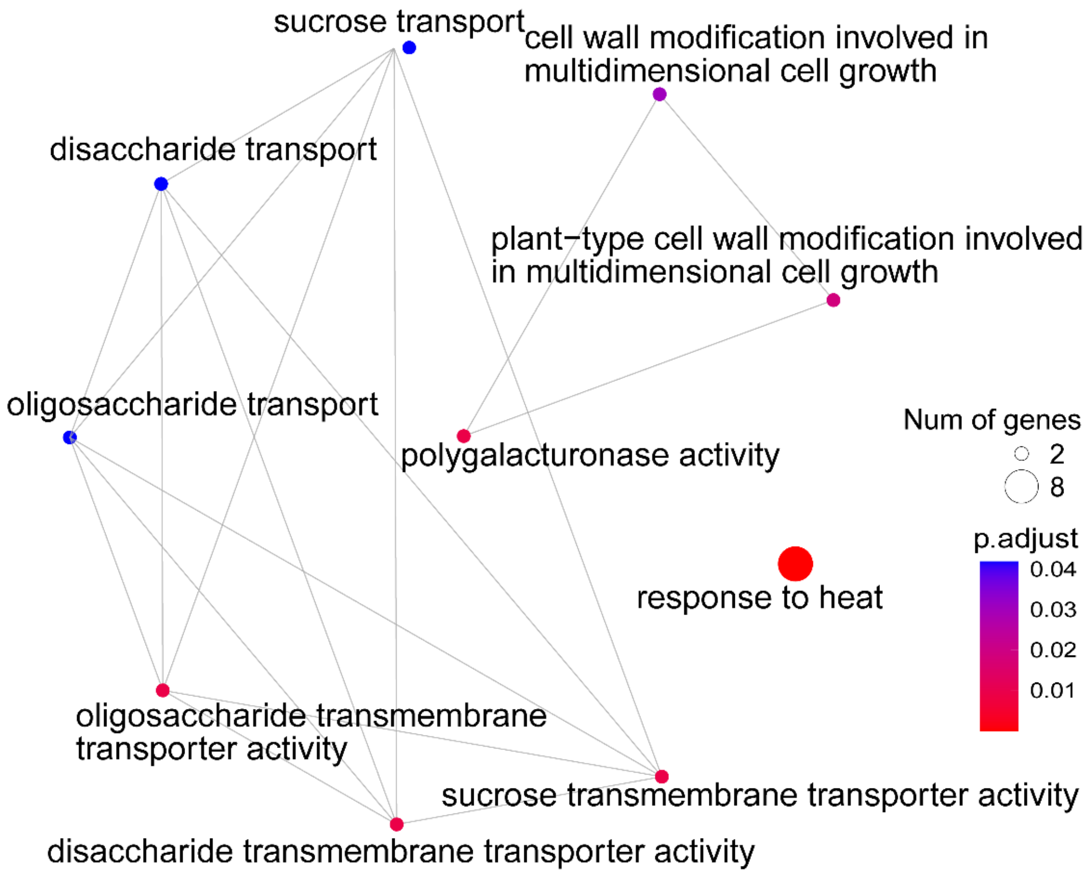

Supplementary Figure 21. GO enrichment of Hub genes in the brown module.

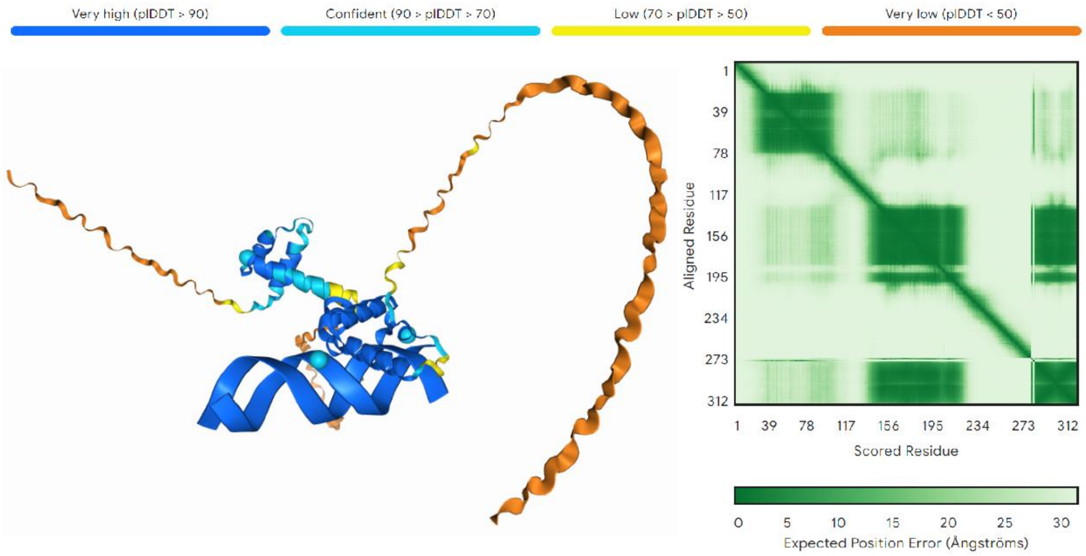

Supplementary Figure 22. illustrates the binding prediction of PaR2R3-MYB to the *PaSUC1.1* candidate promoter using AlphaFold3. The MYB protein sequence and a 19 bp double-stranded DNA fragment containing the MBS element (CAACTG) were entered separately at alphafoldserver (https://alphafoldserver.com/) for binding prediction.

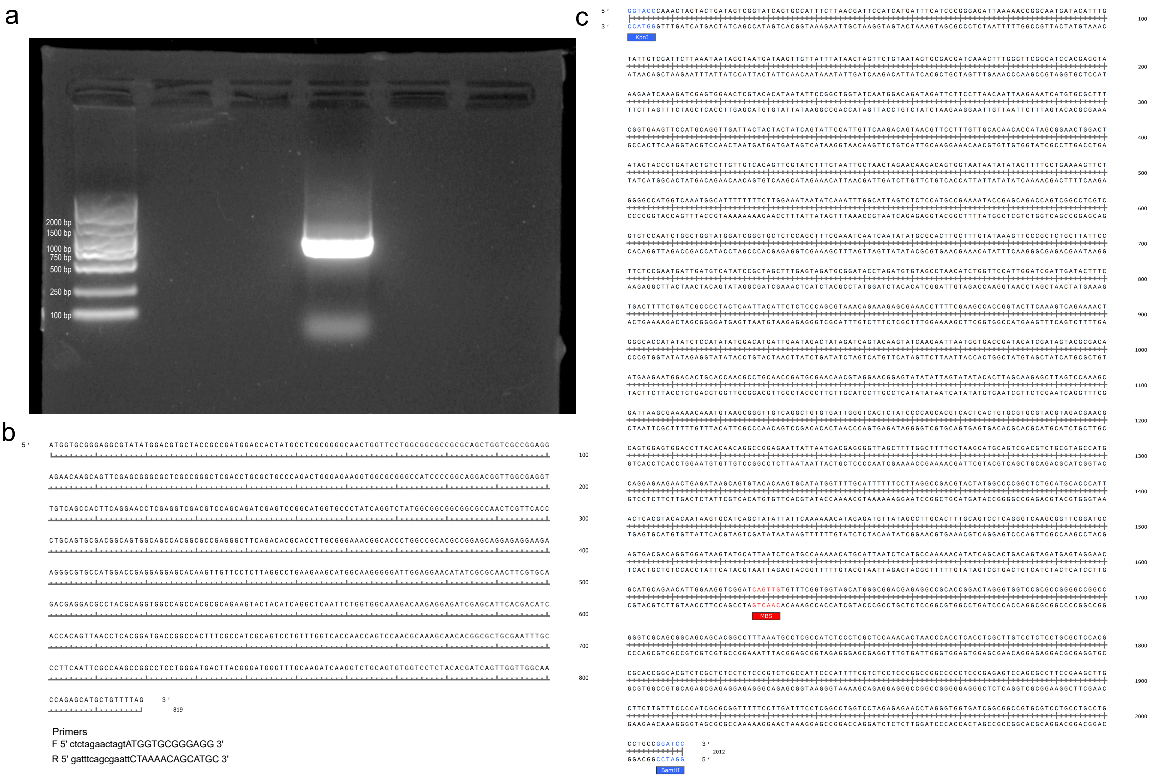

Supplementary Figure 23. Cloning and analysis of PaR2R3-MYB and *PaSUC1.1* candidate promoters. a. Electropherogram of cloned *PaR2R3-MYB* gene from total cDNA library. b. *PaMYB* nucleotide sequence after identification by Sanger sequencing. c. Nucleotide sequence of the *PaSUC1.1* promoter. Blue markers on both ends are added endonuclease sequences. The predicted MBS element (CAACTG) is marked in red.

Supplementary Figure 24: Uncropped and unedited gel images.

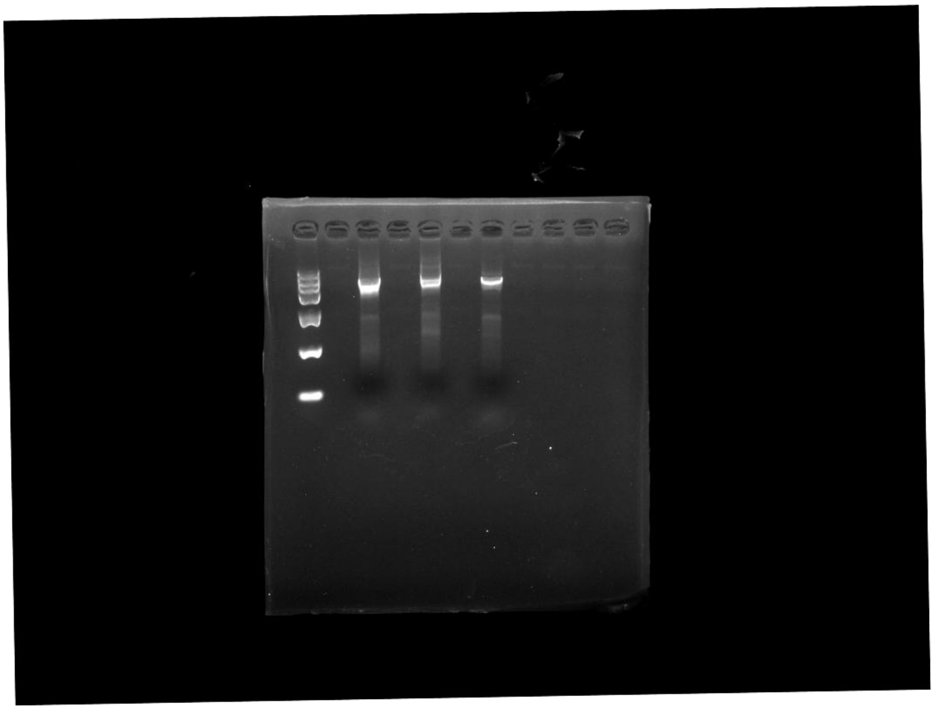

Uncropped and unedited gel images Supplementary Figure 12b

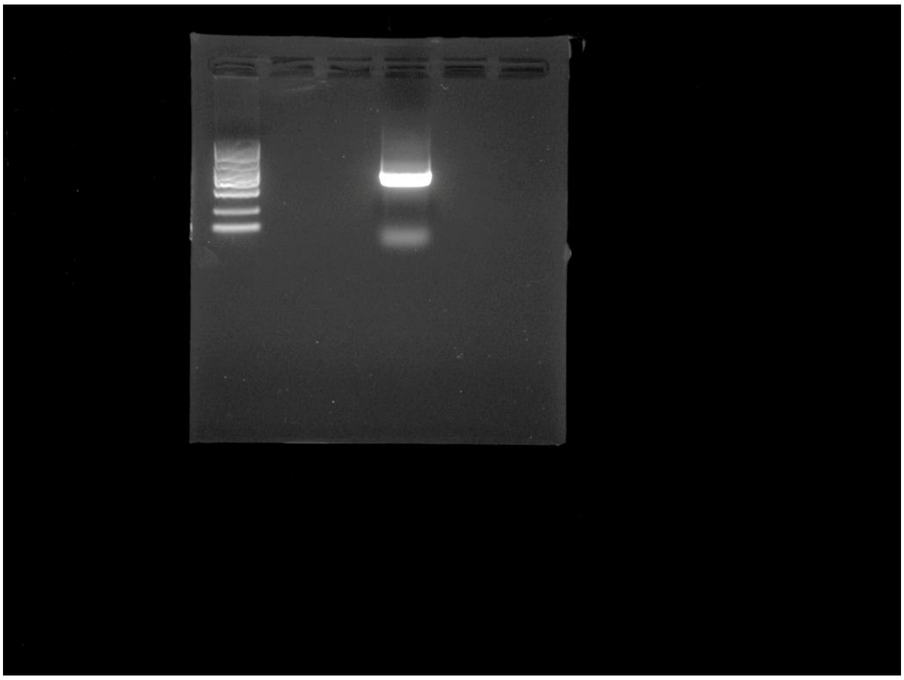

Uncropped and unedited gel images Supplementary Figure 20a

Supplementary Table 1 *P. australis* genome Hi-C assisted assembly statistics.

	Total (bp)	Contig Number	Contigs N50 (bp)	Scaffold Number	Scaffold N50 (bp)
Primary assembly	874,619,212	312	33,936,801	-	-
Hi-C assisted pre-assembly	852,116,256	79	34,052,747	-	-
Chromosomes after Hi-C-assisted assembly	847,498,707	27	34,052,747	25	34,052,747

Supplementary Table 2 Telomere Repeat Unit Finding Statistics.

canonical repeat unit	count	canonical repeat unit	count	canonical repeat unit	count
AAACCCT	40863	AACCC	9127	AAACC	1582
AAGAGAAGAG	25960	AAGAG	8398	ACCCT	1116
AAAGAAAG	16600	AGGGG	4796	AAACCTAAACCT	1018
AAAACCCT	16307	ACATCCTG	4752	AAACCT	997
AAAAG	16122	CCCCGGG	4606	AAACCCAACCCT	942
AACTC	13178	AGATAGAT	3734	AGAGCC	844
AACCT	13151	AAGAC	3690	CCGCG	760
AACCCT	11364	AAACCCCT	1929	AGCGG	726
AAAAACCCT	10192	AACCTG	1608		

Supplementary Table 3 Genomic statistics of *P. australis* at chromosome level.

Chr	Superscaffolds	Length (bp)	Num of contigs	Num of genes	GC %	QV	Telomere status	Num of repetitions on the left	Num of repetitions on the right
1A	8	20,193,544	1	953	44.45%	48.08	both	1438	1975
1B	7	25,746,373	1	1076	44.02%	48.79	both	709	1291
2A	16	23,702,067	1	1008	43.98%	47.41	both	1889	1106
2B	15	24,664,423	1	1110	44.08%	48.26	right	0	781
3A	2	24,084,666	1	956	43.91%	48.99	both	1103	3221
3B	1	33,936,801	1	1159	44.15%	48.2	both	2020	1697
4A	24	25,221,131	1	1132	45.14%	49.34	left	2008	0
4B	23	27,472,612	1	1116	45.12%	48.15	left	1194	0
5A	18	28,937,882	1	1340	44.12%	47.17	left	1571	0
5B	17	31,241,098	1	1431	44.05%	48.33	both	2286	503
6A	22	30,183,679	1	1459	43.99%	47.47	both	543	2419
6B	21	30,499,992	2	1512	44.41%	48.36	both	451	2801
7A	14	31,468,791	1	1815	44.07%	47.98	both	1197	1492
7B	13	37,131,324	1	1849	44.39%	49.03	both	1464	1433
8A	10	33,085,515	1	1649	44.11%	47.91	both	801	2315
8B	9	37,281,543	1	1702	44.11%	49.74	both	2879	1689
9A	4	34,052,747	1	1746	44.20%	48.79	both	1669	1327
9B	3	35,764,372	1	1590	44.15%	49.59	both	2384	2130
10A	12	43,967,752	1	2434	44.09%	48.58	both	1119	2073
10B	11	44,949,043	1	2304	44.02%	49.77	both	905	469
11A	5	49,387,842	1	2881	44.19%	49.22	left	1662	0
11B	6	48,350,371	1	2642	43.92%	48.59	both	1604	1041
12A	19	54,964,116	1	2917	44.05%	49.1	both	1703	2128
12B	20	50,013,265	1	2934	44.03%	48.37	both	2228	1509
B	25	21,198,758	2	277	44.73%	57.28	left	3067	0

Note: QV (Quality Value): the quality value of the bases, for example, Q30 means 99.9% consistency accuracy, Q40 is 99.99%.

Supplementary Table 4 Survey and full-length transcriptome data mapping statistics.

Data type	Mapping rate (%)	Average sequencing depth	Coverage (%)	Coverage (>= 5X, %)	Coverage (>= 10X, %)	Coverage (>= 20X, %)
Survey data	98.62	54.4	99.91	99.78	99.5	97.67
Iso_Seq data	99.99	36.65	99.99	99.91	99.62	92.27

Supplementary Table 5 Genomic SNP and Indel detection.

	SNP			Indel		
	Number	Percentage of indel (%)	Percentage of genome (%)	Number	Percentage of indel (%)	Percentage of genome (%)
All	8,240,383	100.00	0.9670	477,839	100.00	0.0561
Heterozygosis	8,234,289	99.93	0.9663	475,777	99.57	0.0558
Homology	6,094	0.07	0.0007	2,062	0.43	0.0002

Supplementary Table 6: Genetic Characteristics Statistics of Poaceae Species.

Species	Number of genes	Average Gene Length (bp)	Average Exon Length (bp)	Average Intron Length (bp)	Number of introns	Number of genes with introns	The proportion of genes with introns
Phragmites australis	41008	4758.44	301.18	720.66	177546	36871	89.91%
Sorghum bicolor	31697	3943.00	341.03	1159.11	136957	26248	82.81%
Setaria viridis	32252	3502.05	345.89	561.50	139740	26452	82.02%
Brachypodium distachyon	30000	3644.30	346.66	484.87	131832	24639	82.13%
Oryza sativa	33618	3759.88	359.11	755.31	142156	27134	80.71%
Cleistogenes songorica	55440	2694.08	237.85	408.83	210573	41729	75.27%
Dendrocalamus latiflorus Munro	135231	4329.14	286.67	818.72	494549	109935	81.29%
Panicum hallii	29363	3547.66	317.23	1585.00	133954	24037	81.86%
Pennisetum purpureum Schum	65485	4093.50	243.44	647.83	282880	53475	81.66%
Panicum virgatum	68425	3817.89	329.93	1751.62	295572	56689	82.85%
Setaria italica	30123	3554.79	332.35	1218.58	136204	24963	82.87%
Saccharum spontaneum	83826	3765.88	265.44	578.26	347776	70785	84.44%
Triticum aestivum	136398	3754.45	366.15	5252.96	562185	108198	79.33%
Zea mays	44703	4347.27	328.09	2802.13	183738	34650	77.51%
Aegilops tauschii	57065	3739.08	371.31	8542.50	195239	42916	75.21%

Supplementary Table 7 Sequences of three overlapping primers designed within the high-density region of *P. australis* Chr25 chromosome tRNA.

D1-F	TTCGTTTGGACCGACGAAGT
D1-R	AGGAGCGTCCGGTGTAATTC
D2-F	CCCGCATCAGGCACTAATCT
D2-R	CAACCTTTTGCGTCCACCTG
D3-F	TCGACCCAGACATAGACGGT
D3-R	GTTTTCGTTGCAAACCCCGA

Supplementary Table 8 Genomic Synteny depth statistics.

	Number of syntenic blocks	Number of collinear genes pairs
P. australis vs *O. sativa*	246	19384
P. australis vs *C. songorica*	614	25732
P. australis A vs *O. sativa*	87	14311
P. australis B vs *O. sativa*	93	13972
P. australis A vs *C. songorica*	216	19090
P. australis B vs *C. songorica*	241	18727

Supplementary Table 9 Cis-acting element statistics for the promoter of *PaSUC1.1* (rna-Pau34219.1).

Gene ID	Name of cis acting element	Cis acting element motif	Functional classification of cis acting elements
rna-Pau34219.1	TC-rich repeats	GTTTTCTTAC	cis-acting element involved in defense and stress responsiveness
rna-Pau34219.1	LTR	CCGAAA	cis-acting element involved in low-temperature responsiveness
rna-Pau34219.1	LTR	CCGAAA	cis-acting element involved in low-temperature responsiveness
rna-Pau34219.1	LTR	CCGAAA	cis-acting element involved in low-temperature responsiveness
rna-Pau34219.1	ABRE	ACGTG	cis-acting element involved in the abscisic acid responsiveness
rna-Pau34219.1	ABRE	ACGTG	cis-acting element involved in the abscisic acid responsiveness
rna-Pau34219.1	ABRE	ACGTG	cis-acting element involved in the abscisic acid responsiveness
rna-Pau34219.1	ARE	AAACCA	cis-acting regulatory element essential for the anaerobic induction
rna-Pau34219.1	G-box	CACGAC	cis-acting regulatory element involved in light responsiveness
rna-Pau34219.1	G-box	CACGTC	cis-acting regulatory element involved in light responsiveness
rna-Pau34219.1	G-box	TACGTG	cis-acting regulatory element involved in light responsiveness
rna-Pau34219.1	G-box	CACGTC	cis-acting regulatory element involved in light responsiveness
rna-Pau34219.1	CGTCA-motif	CGTCA	cis-acting regulatory element involved in the MeJA-responsiveness
rna-Pau34219.1	CGTCA-motif	CGTCA	cis-acting regulatory element involved in the MeJA-responsiveness
rna-Pau34219.1	CGTCA-motif	CGTCA	cis-acting regulatory element involved in the MeJA-responsiveness
rna-Pau34219.1	TGACG-motif	TGACG	cis-acting regulatory element involved in the MeJA-responsiveness
rna-Pau34219.1	TGACG-motif	TGACG	cis-acting regulatory element involved in the MeJA-responsiveness
rna-Pau34219.1	TGACG-motif	TGACG	cis-acting regulatory element involved in the MeJA-responsiveness
rna-Pau34219.1	Sp1	GGGCGG	light responsive element
rna-Pau34219.1	MBS	CAACTG	MYB binding site involved in drought-inducibility
