# Supplementary material for: Telomere-to-telomere Phragmites australis reference genome assembly with a B chromosome provides insights into its evolution and polysaccharide biosynthesis
Source: Commun Biol. 2025 Jan 17;8:73. doi: 10.1038/s42003-025-07532-y (PMC11742667; doi:10.1038/s42003-025-07532-y)
Supplement: Supplementary file 2 — Reporting Summary [file 42003_2025_7532_MOESM2_ESM.pdf]

nature portfolio

Corresponding author(s): Suxia Cui

Last updated by author(s): Dec 14, 2024

Reporting Summary

Nature Portfolio wishes to improve the reproducibility of the work that we publish. This form provides structure for consistency and transparency in reporting. For further information on Nature Portfolio policies, see our Editorial Policies and the Editorial Policy Checklist.

Statistics

For all statistical analyses, confirm that the following items are present in the figure legend, table legend, main text, or Methods section.

n/a	Confirmed	
☐	☒	The exact sample size (*n*) for each experimental group/condition, given as a discrete number and unit of measurement
☐	☒	A statement on whether measurements were taken from distinct samples or whether the same sample was measured repeatedly
☒	☐	The statistical test(s) used AND whether they are one- or two-sided<br>*Only common tests should be described solely by name; describe more complex techniques in the Methods section.*
☒	☐	A description of all covariates tested
☐	☒	A description of any assumptions or corrections, such as tests of normality and adjustment for multiple comparisons
☐	☒	A full description of the statistical parameters including central tendency (e.g. means) or other basic estimates (e.g. regression coefficient) AND variation (e.g. standard deviation) or associated estimates of uncertainty (e.g. confidence intervals)
☐	☒	For null hypothesis testing, the test statistic (e.g. *F*, *t*, *r*) with confidence intervals, effect sizes, degrees of freedom and *P* value noted<br>*Give P values as exact values whenever suitable.*
☒	☐	For Bayesian analysis, information on the choice of priors and Markov chain Monte Carlo settings
☒	☐	For hierarchical and complex designs, identification of the appropriate level for tests and full reporting of outcomes
☐	☒	Estimates of effect sizes (e.g. Cohen's *d*, Pearson's *r*), indicating how they were calculated

Our web collection on statistics for biologists contains articles on many of the points above.

Software and code

Policy information about availability of computer code

Data collection	No software was used to collect the data.
Data analysis	SOAPnuke v.2.1.0, jellyfish v.2.2.10, genomescope v2.0, smudgeplot, pbccs v.6.4.0, hifiasm v0.16.1, Juicer v.1.6, 3D-DNA v.180922, tidk v.0.2.3, pbmm2 v.1.13.1, pb-CpG-tools v.2.3.2, WGDI v.0.6.1, JCVI v.0.5.7, RAxML v.8.2.12, MCScanX, HMMER v.3.0, BLAST v.2.2.29, quast v.5.2.0, minimap2 v.2.12, Merqury v.1.3, BWA v.0.7.17, GATK v.3.8, BUSCO v.5.2.2, CEGMA v.2.5, LTR_Finder v.1.07, genometools v.1.6.2, LTR_retreiver v.2.9.7, ggplot2, RepeatMasker v.4.1.0, RepeatProteinMask v.4.1.2, MISA v.2.1, Augustus v.3.3, Trf v.4.09, MAKER v.3.0, GlimmerHMM v.3.0.4, PASA v.2.5, RepeatModeler v.2.0.2, tRNAscan-SE v.2.0.12, Infernal v.1.1.4, OrthoFinder2 v.2.5.4, muscle v.3.8.31, r8s v.1.81, PAML v.4.10.0, CAFÉ v.4.2.1, fastp v.0.21.0, HISAT2 v.2.2.1, samtools v.1.13, featureCounts v.2.0.3, WGCNA v.1.72, clusterProfiler v.4.0, Cytoscape v.3.8.2, TBtools v.2.03, GraphPad Prism v.8.0.2, IGV v.2.12.3, Rstudio v.26.2.4, R v.4.2.2, GenomeSyn v 1.2.6

For manuscripts utilizing custom algorithms or software that are central to the research but not yet described in published literature, software must be made available to editors and reviewers. We strongly encourage code deposition in a community repository (e.g. GitHub). See the Nature Portfolio guidelines for submitting code & software for further information.

Data

Policy information about availability of data

All manuscripts must include a data availability statement. This statement should provide the following information, where applicable:

- Accession codes, unique identifiers, or web links for publicly available datasets
- A description of any restrictions on data availability
- For clinical datasets or third party data, please ensure that the statement adheres to our policy

Whole genome and transcriptome raw sequencing data used in this study have been deposited at the National Center for Biotechnology Information (NCBI) under accession number PRJNA1055898. This genome assembly, annotations, protein sequences, CDS sequences, and the numerical source data underlying the graphs and charts are available for download from Figshare (https://doi.org/10.6084/m9.figshare.27016279).

Research involving human participants, their data, or biological material

Policy information about studies with human participants or human data. See also policy information about sex, gender (identity/presentation), and sexual orientation and race, ethnicity and racism.

Reporting on sex and gender	NA
Reporting on race, ethnicity, or other socially relevant groupings	NA
Population characteristics	NA
Recruitment	NA
Ethics oversight	NA

Note that full information on the approval of the study protocol must also be provided in the manuscript.

Field-specific reporting

Please select the one below that is the best fit for your research. If you are not sure, read the appropriate sections before making your selection.

☒ Life sciences ☐ Behavioural & social sciences ☐ Ecological, evolutionary & environmental sciences

For a reference copy of the document with all sections, see nature.com/documents/nr-reporting-summary-flat.pdf

Life sciences study design

All studies must disclose on these points even when the disclosure is negative.

Sample size	Sample size calculation was not performed in this study. Young leaf tissues of biennial Phragmites australis were collected for genome survey sequencing, HiFi sequencing, and Hi-C sequencing. Sample size calculation was not performed in this study. At the same time, different tissues are used for full-length transcriptome sequencing (Nine types of tissues including flower, stem apical meristem, above-ground stems, leaves, above-ground stem buds, rhizome internodal tissues, rhizome nodal meristem, rhizome buds, and fibrous roots) and transcriptome sequencing (including mature leaves, above-ground stems, and rhizome tissues, in three biological replicates) were collected.
Data exclusions	Using SOAPnuke for genome survey dataQuality control and filtering.Raw data from the PacBio Sequel II sequencing platform was used to generate 32.53 Gbps Hifi reads using the PacBio smrt link toolkit.The RNAseq data were filtered for low-quality reads using fastp.
Replication	There are three biological replicates per tissue in transcriptome sequencing.
Randomization	Randomization was not applied in our genomic and transcriptomic analyses.
Blinding	Since all our analyses were performed with known samples, this study does not apply to blinding.

Reporting for specific materials, systems and methods

We require information from authors about some types of materials, experimental systems and methods used in many studies. Here, indicate whether each material, system or method listed is relevant to your study. If you are not sure if a list item applies to your research, read the appropriate section before selecting a response.

Materials & experimental systems

n/a	Involved in the study
☒	☐ Antibodies
☒	☐ Eukaryotic cell lines
☒	☐ Palaeontology and archaeology
☒	☐ Animals and other organisms
☒	☐ Clinical data
☒	☐ Dual use research of concern
☐	☒ Plants

Methods

n/a	Involved in the study
☒	☐ ChIP-seq
☒	☐ Flow cytometry
☒	☐ MRI-based neuroimaging

Dual use research of concern

Policy information about dual use research of concern

Hazards

Could the accidental, deliberate or reckless misuse of agents or technologies generated in the work, or the application of information presented in the manuscript, pose a threat to:

No	Yes
☒	☐ Public health
☒	☐ National security
☒	☐ Crops and/or livestock
☒	☐ Ecosystems
☒	☐ Any other significant area

Experiments of concern

Does the work involve any of these experiments of concern:

No	Yes
☒	☐ Demonstrate how to render a vaccine ineffective
☒	☐ Confer resistance to therapeutically useful antibiotics or antiviral agents
☒	☐ Enhance the virulence of a pathogen or render a nonpathogen virulent
☒	☐ Increase transmissibility of a pathogen
☒	☐ Alter the host range of a pathogen
☒	☐ Enable evasion of diagnostic/detection modalities
☒	☐ Enable the weaponization of a biological agent or toxin
☒	☐ Any other potentially harmful combination of experiments and agents

Plants

Seed stocks	The samples were taken from two-year old reeds in the reed planting sample site of Capital Normal University. The sampling time was June 2022.
Novel plant genotypes	All the samples were wild types.
Authentication	NA
